# A Noval Established Cuproptosis-Associated LncRNA Signature for Prognosis Prediction in Primary Hepatic Carcinoma

**DOI:** 10.1155/2022/2075638

**Published:** 2022-09-15

**Authors:** Lan Luo, Xiaoyan Hu, Aoshuang Huang, Xiaofang Liu, Lingyun Wang, Tao Du, Lei Liu, Ming Li

**Affiliations:** Department of Hematology and Oncology, The First People's Hospital of Guiyang, No. 97, Boai Road, Nanming, Guiyang, Guizhou 550002, China

## Abstract

The copper ion content in the body maintains homeostasis, and when dysregulated, it can produce cytotoxicity and induce cell death through a variety of pathways. Cuproptosis refers to copper ions combining directly with acylated molecules, leading to the accumulation of oligomerization of lipoylated protein and subsequent downregulation of iron-sulfur cluster proteins; this induces proteotoxic stress and cell death. This study on the relationship between cuproptosis-related lncRNAs (CRLns) and the prognosis of primary hepatic carcinoma (PHC) has important clinical guiding significance for the diagnosis and treatment of PHC. Prognosis-related CRLRs were identified via rank-sum tests, correlational analyses, and univariate Cox regression, and a CRLR risk-scoring model (CRLRSM) was constructed using LASSO Cox regression. Patients were divided into high-risk and low-risk groups based on the median CRLRSM scores. Variance analysis for cuproptosis-related genes, gene set enrichment analysis, and correlational analysis for risk and immunity were performed using boxplots. Quantitative polymerase chain reactions were used to verify the CRLR levels in PHC cell lines. The study results showed that patients in the CRLRSM high-risk group had worse survival rates than those in the low-risk group. The PHC stage and risk score were independent prognostic factors for hepatocellular carcinoma. There were 7 CRLRs (MIR210HG, AC099850.3, AL031985.3, AC012073.1, MKLN1-AS, KDM4A-AS1, and PLBD1-AS1) associated with PHC prognosis, primarily through cellular metabolism, growth, proliferation, apoptosis, and immunity. In conclusion, the overexpression of 7 CRLRs in patients with PHC indicates a poor prognosis.

## 1. Introduction

Copper ion is an essential metal element for bacteria, animals, and humans, and an indispensable cofactor in the process of life activities [1]. Under normal physiological conditions, copper ions maintain a low concentration in organisms and maintain dynamic balance, and excessive accumulation of copper ions induces cell death [2]. The mechanism is unknown, but in recent years, it has been found that copper ions combine with anticancer drugs to produce reactive oxygen species, form oxidative stress, inhibit antiapoptotic factors, activate apoptosis-related pathways, and thus induce apoptosis of cancer cells [3, 4]. Cu^2+^ complexes can be potent proteasome inhibitors, inhibiting proteasome activity in some tumors and subsequently inhibiting cell proliferation [5, 6]. Recently, Tsvetkov et al. found that copper ions directly bind to the lipoacylated component of the tricarboxylic acid cycle (TCA) in mitochondrial respiration, leading to the aggregation of lipoacylated proteins and subsequent downregulation of iron-sulfur cluster proteins, leading to proteotoxic stress and ultimately cell death. Copper carriers were also found to kill specific drug-resistant cancer cells. The research team identified this as a new type of cell death, termed cuproptosis [7].

Primary hepatic carcinoma (PHC) is one of the most common digestive system malignancies and comprises mainly hepatocellular carcinoma (HCC) and intrahepatic cholangiocarcinoma (ICC). The pathogenesis of PHC is a complex and multistage process, involving epigenetics and genetics, finally leading to the cancerization of hepatic cells. Although ever-improving surgical approaches and emerging drugs have lengthened patient survival, their overall outcomes remain unsatisfactory. The five-year survival rate in patients with PHC is merely 20% [8].

In order to prolong the survival of patients with Primary hepatic carcinoma, we identified FDX1, LIAS, LIPT1, DLD, DLAT, PDHA1, PDHB, MTF1, GLS, and CDKN2A as cuproptosis-related genes [7] through previous studies. Long noncoding RNAs (lncRNAs) are nonbiased RNAs with a length of more than 200 nucleotides [9]. Accumulating evidence shows that lncRNA plays a complex and precise regulatory role in the process of tumorigenesis and development by acting as oncogenes or tumor performing factors [10–13]. They can not only regulate the proliferation, differentiation, invasion, and metastasis of cancer cells but also regulate the metabolic reprogramming of cancer cells [14–16]. There are currently few studies regarding the correlation between cuproptosis-related lncRNAs and PHC. Therefore, we built a new model of cuproptosis-related lncRNAs to predict the prognoses of PHC patients, assess the predictive performance of these lncRNAs, interpret individual differences, identify targets, and improve survival, which would be of great value in clinical practice.

## 2. Materials and Methods

### 2.1. Collection of Gene Expression and Clinical Data

There were 421 sets of public RNA-sequencing transcriptome data and clinical data extracted from UCSC Xena (http://xena.ucsc.edu/); one set of clear cell adenocarcinomas and six sets with unavailable clinical information were excluded. Thus, 414 sets were included: 364 sets of cancer tissue samples and 50 sets of normal tissue samples. HCC and HCC concomitant with ICC were the primary pathological types. The collected clinical characteristics included age, sex, pathological stage, overall cancer stage, survival state, and overall survival (OS) of the patients. Ten cuproptosis-related genes were identified through screening of genome-wide CRISPR–Cas9 deletions [7].

### 2.2. Cuproptosis-Related lncRNAs

There were 19266 mRNAs and 13431 lncRNAs that were recognized from the cancer tissues and normal tissues using the “Perl” software, and 10 cuproptosis-related genes were identified through screening of genome-wide CRISPR–Cas9 deletions. To assess lncRNAs associated with cuproptosis, Spearman's rank correlation coefficient was analyzed using the “limma” R package. The lncRNAs with *p* < 0.001 and a correlation coefficient of 0.4 were selected as cuproptosis-related lncRNAs. Univariate Cox regression was used to identify 106 prognosis-related lncRNAs (*p* < 0.05).

### 2.3. Construction of the Prediction Model

The Least Absolute Shrinkage and Selection Operator (LASSO) was processed using the “glmnet” R package to sort out cuproptosis-related lncRNAs (CRLRs), which were then plugged into a multivariate Cox model to construct a CRLR risk-scoring model (CRLRSM). The following formula was used for the risk scoring:

(1) $$\begin{array}{c} \text{Risk score}=\sum {\text{Coef}}_{\text{lncRNAs}}\times {\text{Exp}}_{\text{lncRNAs}}. \end{array}$$

Patients were divided into high-risk and low-risk groups based on the median CRLRSM scores.

### 2.4. Survival Analysis and Model Validation

A total of 364 PHC patients were assigned in a 5 : 5 ratio to a training (*n* = 184) or test group (*n* = 180) for validation of the risk score. Kaplan–Meier analysis was performed, and the area under the receiver operator characteristic (ROC) curve (AUC) was calculated using R (version 4.1.3) for the three groups of low-risk and high-risk patients to estimate the accuracy and sensitivity of the OS prediction. Principal components analysis was used to evaluate differences in the expression of CRLRs in patients with PHC. To evaluate whether the risk score would be an independent prognostic factor and to identify clinical characteristics that could be regarded as independent prognostic factors, ROC univariate analysis, and Cox multivariate analysis were performed for clinical characteristics (age, sex, pathological stage, and overall stage) and the risk score. The ROC was used to compare the predictive performance between the clinical characteristics and risk score.

### 2.5. Nomogram Predicted Survival

A predictive nomogram for cancer risk can estimate the survival of specific cancer patients based on individual data, and they are of great value in clinical practice [17]. Using the “rms” and “Hmisc” R packages, we plotted a nomogram that included stages and risk scores. The AUC and calibration charts were used to evaluate the predictive accuracy and value of the 1-year, 3-year, and 5-year survival rates. A consistency index (C-index) was used to validate the performance of the nomogram predictions.

### 2.6. Variance Analysis for Cuproptosis-Related Genes

A boxplot is used to visualize the dispersion of data, which can be used to compare the distribution characteristics of multiple sets of data. Therefore, we constructed a boxplot, using the “limma” and “ggpubr” packages in R to evaluate whether there was a difference in the expression of cuproptosis-related genes between high-risk and low-risk groups.

### 2.7. Correlational Analysis for Risk and Immunity

The types and distributions of immune cells from the 414 patients were analyzed using the CIBERSORT algorithm, and the relationship between the risk score and the amount of immune cell invasion was assessed using Spearman's correlation analysis. Statistical significance was indicated by a *p* value < 0.05.

### 2.8. Gene Set Enrichment Analysis

The patients were assigned to the low-risk or high-risk group based on the prediction model. Gene set enrichment analysis was performed to identify significantly associated biological functions and signaling pathways, which might be potential pathways related to CRLR regulation. Statistical significance was indicated by a false discovery rate (FDR) < 0.05.

### 2.9. Cell Culturing

Human cell lines of normal hepatocytes (LO-2) and hepatomas (Huh7, Hep3B, and SK-Hep-1) were purchased from the Cell Collection Center of the Chinese Academy of Sciences. All cell lines were incubated in 5% CO2 at 37°C on a medium supplemented with 10% fetal bovine serum and 1% penicillin–streptomycin. The LO-2 cells were cultured using RPMI1630 medium, Huh7 cells were cultured using DMEM high-glucose medium, and Hep3B and SK-Hep-1 cells were cultured using MEM high-glucose medium.

### 2.10. Real-Time Polymerase Chain Reaction (PCR)

RNA was extracted from 1 × 10^6^ LO-2, Huh7, Hep3B, and SK-Hep-1 cells using TRIzol reagent, followed by reverse-transcription to synthesize cDNA using a cDNA synthesis kit according to the manufacturer's instructions. Based on the SYBR-Green method (TaKaRa), the 7500 Real-Time PCR System (Applied Biosystems) was used to measure the levels of the resulting cDNA. The reaction program was 30 s at 94°C, followed by 40 cycles of 5 s at 94°C and 35 s at 61°C. The primer sequences are shown in Table 1. Differences in the levels of targeted genes between the test and control groups were analyzed via the 2^−∆∆CT^ method, using the following calculation: △Ct = Ct_target gene_ − Ct_internal reference_. The average △Ct of the control group was recorded as the △Ct_control average_. Subtracting the △Ct_control average_ from the △Ct of each group yields the △△Ct value, that is, △△Ct = △Ct_sample_ − △Ct_control average_. The 2^−∆∆CT^ value of each group, indicating the relative expression of the genes in the group, was then calculated.

### 2.11. Data Analysis

PERL (version 5.32.1.1, https://strawberryperl.com/) was used for gene name translation, lncRNA identification, data-file expression, and phenotyping. All statistical analyses were performed using R software (version 4.1.3) or GraphPad Prism5 (GraphPad Software Inc., La Jolla, CA, USA). A *p* value < 0.05 indicated statistical significance. All experiments were performed twice.

## 3. Results

### 3.1. Data Processing

**Figure 1:**
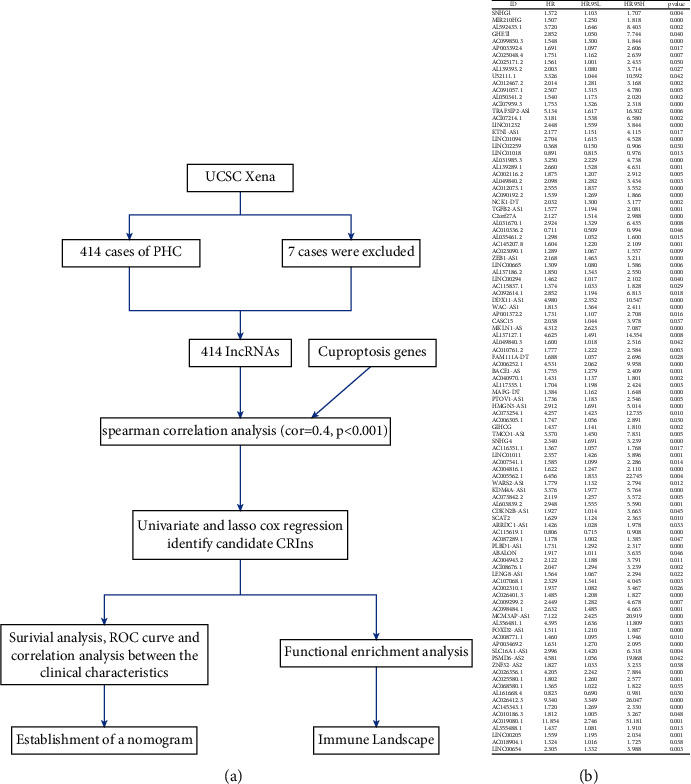
(a) Flow chart of the study. (b) Univariate Cox results for prognosis-associated lncRNA.

**Figure 2:**
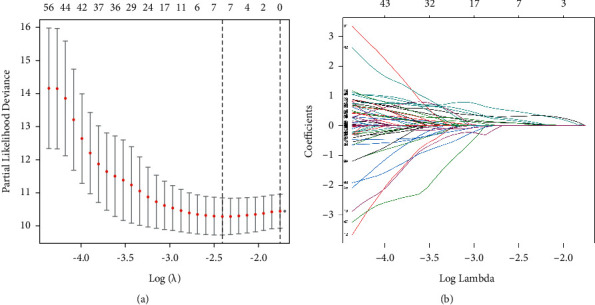
(a) LASSO cross-validation plot based on seven CRLRs. (b) LASSO coefficient of the seven CRLRs in PHC.

There were 414 cases of HCC or HCC with ICC downloaded from UCSC Xena; of these, 50 were pericarcinomatous tissues. A total of 13431 lncRNA were obtained, and 10 cuproptosis-related genes were identified through screening of genome-wide CRISPR–Cas9 deletions (Figure 1(a)). A total of 106 prognosis-related lncRNAs were identified using univariate Cox regression analysis. Seven CRLRs were identified using LASSO Cox regression: MIR210HG, AC099850.3, AL031985.3, C012073.1, MKLN1-AS, KDM4A-AS1, and PLBD1-AS1 (Figures 2(a) and 2(b)).

### 3.2. Construction and Validation of CRLR Risk Model for PHC

Seven CRLRs were identified via LASSO Cox regression using the following risk-scoring formula:

(2) $$\begin{array}{c} \text{Risk score}=\left(\text{MIR}210\text{HG}\times 0.068\right)+\left(\text{AC}099850.3\times 0.017\right)+\left(\text{AL}031985.3\times 0.327\right)+\left(\text{AC}012073.1\times 0.102\right)\\ +\left(\text{MKLN}1-\text{AS}\times 0.426\right)+\left(\text{KDM}4\text{A}-\text{AS}1\times 0.152\right)+\left(\text{KDM}4\text{A}-\text{AS}1\times 0.049\right). \end{array}$$

**Figure 3:**
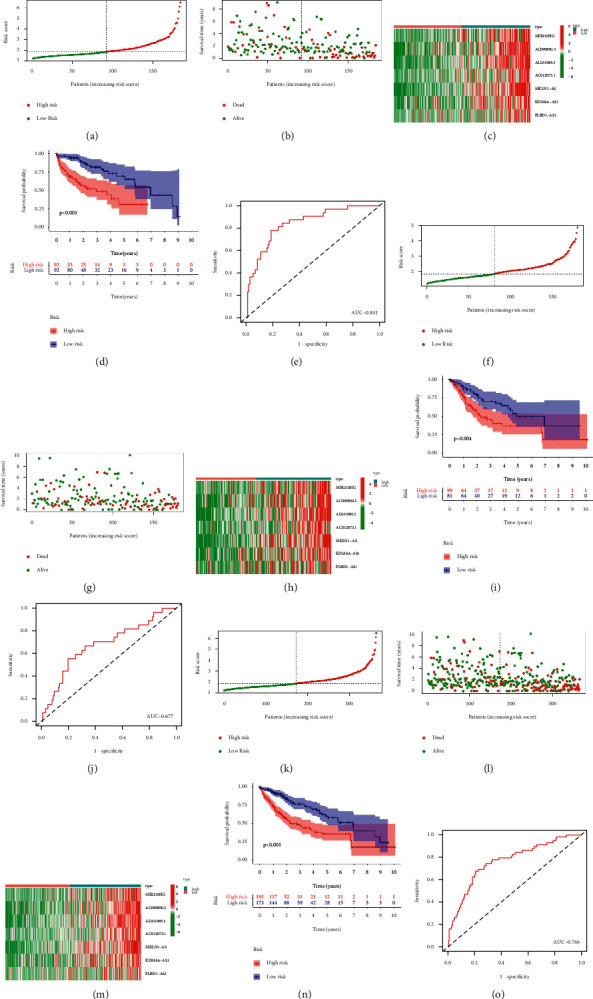
(a) Distribution of risk scores in the training cohort of patients with PHC, based on CRLRs. (b) Scatter plots show the association between the OS and the risk score in the training cohort of PHC patients, according to prognostic features of CRLRs. (c) A heat map shows seven CRLRs (MIR210HG, AC099850.3, AL031985.3, AC012073.1, MKLN1-AS, KDM4A-AS1, and PLBD1-AS1) with high expression in high-risk patients in the training cohort. (d) KM survival curve analysis of the training cohort. (e) Area under the ROC curve for the training cohort, based on CRLR-based prognostic features at 12 months. (f) Distribution of risk scores in the testing cohort of patients with PHC, based on CRLRs. (g) Scatter plots show the association between the OS and the risk score in the testing cohort of PHC patients, according to prognostic features of CRLRs. (h) A heat map shows the same seven CRLRs with high expression in high-risk patients in the testing cohort. (i) KM survival curve analysis of the testing cohort. (j) Area under the ROC curve for the testing cohort, based on CRLRs-based prognostic features at 12 months. (k) Distribution of risk scores for the entire cohort of patients with PHC, based on CRLRs. (l) Scatter plots show the association between the OS and the risk score for the entire cohort of PHC patients, according to prognostic features of CRLRs. (m) Heat map shows the same seven CRLRs with high expression in high-risk patients among the entire cohort. (n) KM survival curve analysis of the entire cohort. (o) Area under the ROC curve for the entire cohort, based on CRLRs-based prognostic features at 12 months.

The patients were divided in a 5 : 5 ratio into training and test groups, based on the median scores of the CRLRSM (Figures 3(a), 3(f), and 3(k)). The whole group is the sum of patients in the training and test groups. The number of deaths increased as the risk score increased (Figures 3(b), 3(g), and 3(l)). A heat map showed that the 7 CRLR genes were related to a poor prognosis (Figures 3(c), 3(h), and 3(m)). There was a significant difference in survival between the high- and low-risk groups in the training group; patients in the low-risk group had longer survival times compared with those in the high-risk group. In the test group as well, patients in the low-risk group had longer survival times than those in the high-risk group (Figure 3(i), *p*=0.004). Likewise, among the whole group, patients in the high-risk group had worse survival times than those in the low-risk group (Figure 3(n), *p* < 0.001).

**Figure 4:**
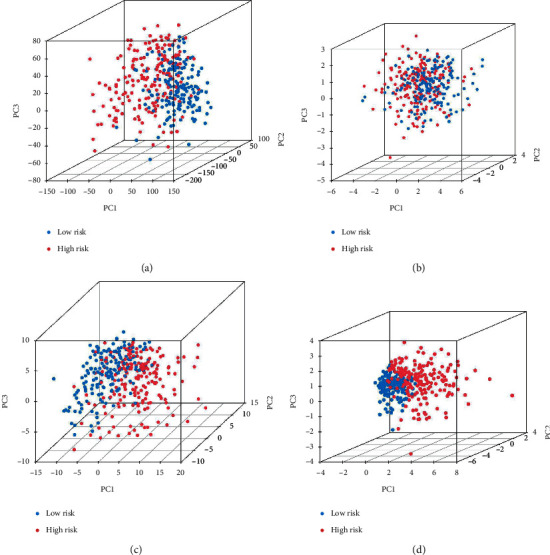
The high-risk and low-risk groups are analyzed using (a) the whole gene set, (b) the cuproptosis gene set, (c) the cuproptosis lncRNA set, and (d) the cuproptosis risk lncRNA set.

**Figure 5:**
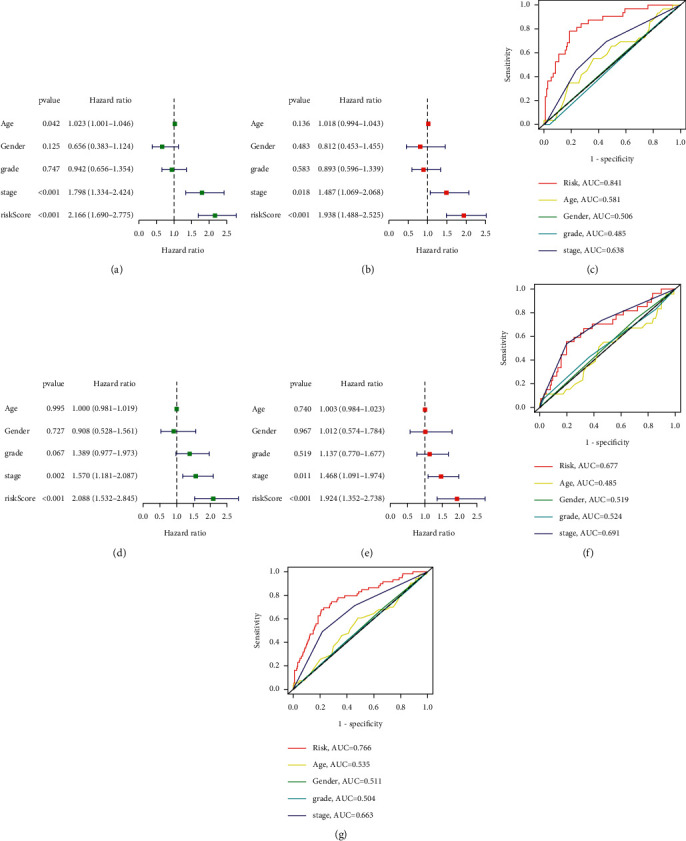
(a) Univariate Cox regression showing that the age, stage, and risk score are associated with OS in the training cohort (*p* < 0.05). (b) Multivariate Cox regression shows that the stage and risk score (*p* < 0.05) are independent prognostic indicators of OS in patients with PHC in the training cohort. (c) ROC curve shows that the stage and risk score have the highest prognostic accuracy in the training cohort. (d) Univariate Cox regression shows that the stage and risk score are associated with OS in the testing cohort (*p* < 0.01). (e) Multivariate Cox regression shows that the stage and risk score (*p* < 0.05) are independent prognostic indicators of OS in patients with PHC in the testing cohort. (f) ROC curve shows that the stage and risk score have the highest prognostic accuracy in the testing cohort. (g) ROC curve shows that the stage and risk score have the highest prognostic accuracy for the entire cohort.

A ROC curve was plotted to predict the 1-year survival and assess the accuracy and sensitivity of the prediction model. The AUC was 0.814 in the training group, 0.677 in the test group, and 0.766 in the whole group, indicating excellent accuracy for the model (Figures 3(e), 3(j), and 3(o)). Principal components analysis confirmed the differential expression of CRLRs in PHC patients (Figures 4(a)–4(d)). To compare the effects of the clinical characteristics and risk score on prognosis, univariate Cox proportional regression was performed. This showed that the overall PHC stage and risk score were correlated with the prognosis of PHC in both the training group (Figure 5(a), *p* < 0.001) and the test group (Figure 5(d), (*p* < 0.01). Multivariate Cox regression analysis showed that the overall stage and risk score in both the training and test groups were independent factors for the prognosis of PHC (Figures 5(b) and 5(e), *p* < 0.05); the risk score was the most significant. A ROC curve was drawn according to the clinical characteristics and risk score, and it showed that the AUC of the risk score was maximized in the training, test, and whole groups; this indicates that the CRLRSM had high accuracy and sensitivity for predicting the prognoses of PHC patients (Figures 5(c), 5(f), and 5(g)).

### 3.3. Predictive Nomogram

**Figure 6:**
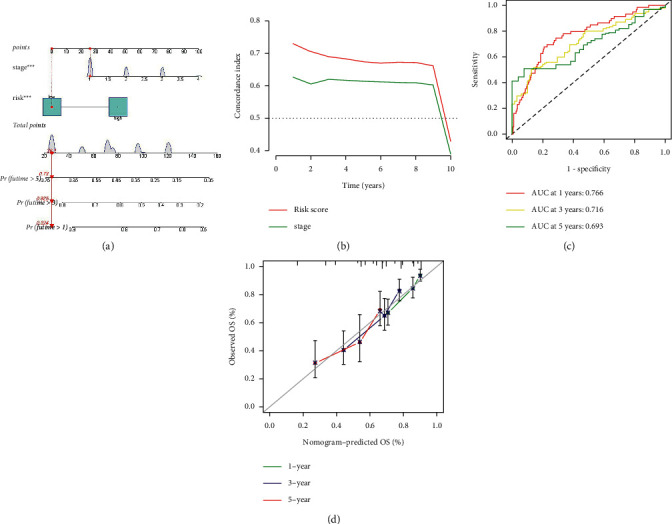
(a) Survival nomogram on risk score and stage. (b) Concordance index on risk score and stage. (c) ROC curve comparing the prognostic power of the nomogram at 1, 3, and 5 years for the entire cohort. (d) Calibration curves were corrected for predicting liver cancer patients at 1, 3, and 5 years for the entire cohort.

A predictive nomogram for cancer risk can estimate the survival of specific cancer patients based on individual data Based on multivariate Cox regression, the overall stage and risk score were included to construct the nomogram (Figure 6(a)). The whole group was internally validated using the C-index (Figure 6(b)). The 1-year, 3-year, and 5-year AUCs of the nomogram were 0.766, 0.716, and 0.693, respectively, suggesting that the nomogram had excellent specificity and sensitivity for predicting the OS time (Figure 6(c)). The calibrated nomogram was consistent with the diagonal, indicating the predictive value of the nomogram for 1-year, 3-year, and 5-year OS (Figure 6(d)). These results demonstrated that the CRLRSM-based nomogram had good prediction performance for the prognoses of patients with PHC.

### 3.4. Enrichment Analysis of Function Pathway Sets

**Figure 7:**
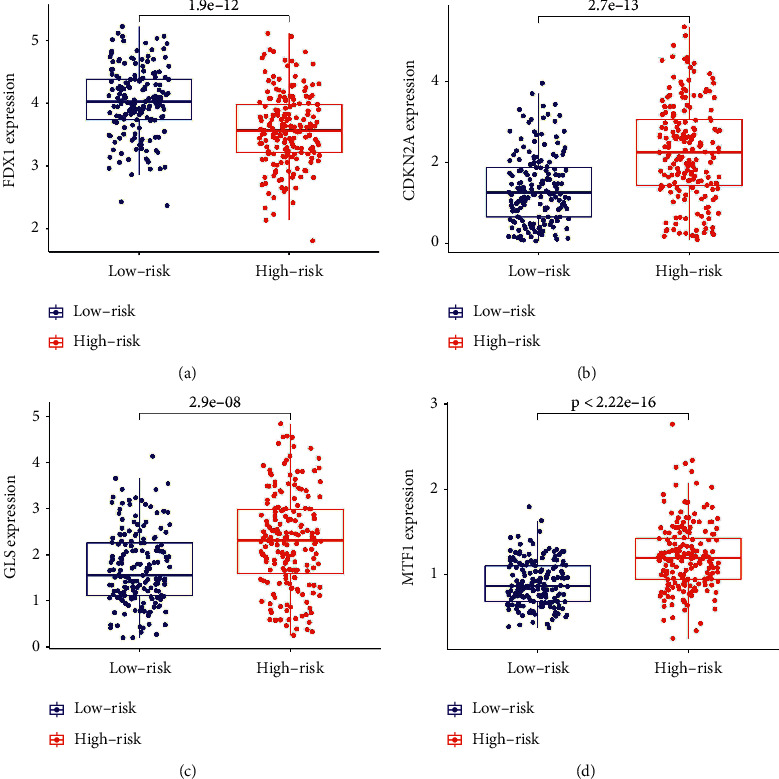
Different expression of (a) FDX1, (b) CDKN2A, (c) GLS, and (d) MTF1 in the high- and low-risk groups.

**Figure 8:**
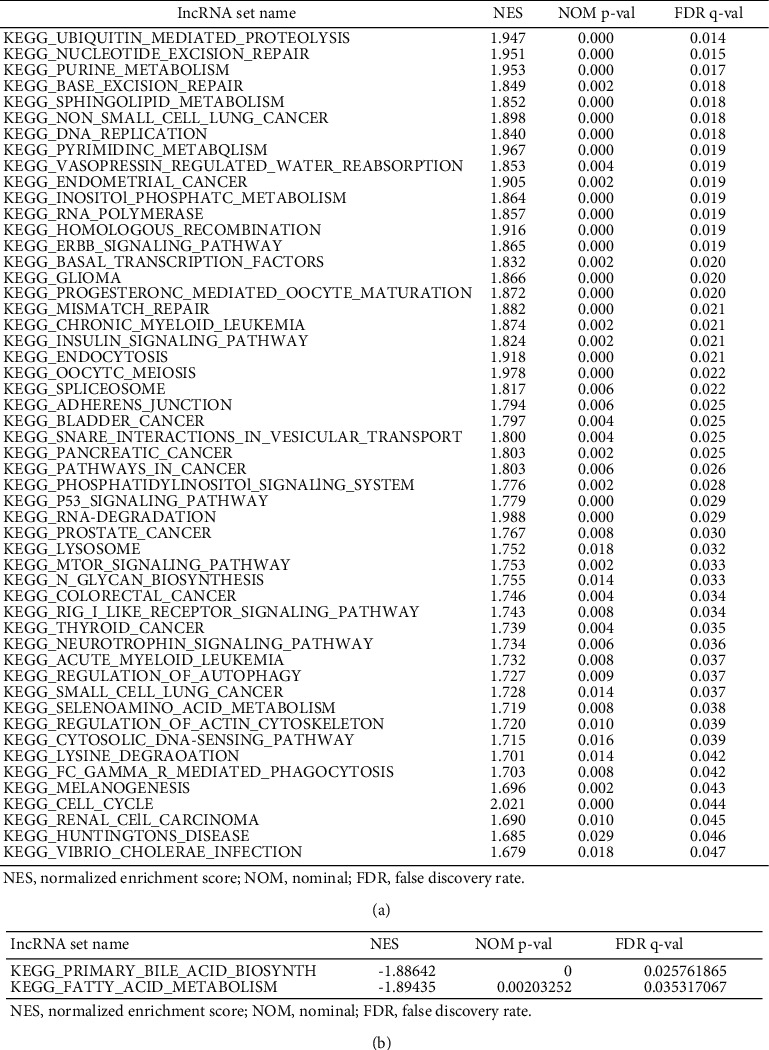
CRLRs set enrichment analysis for the (a) high-risk group and (b) low-risk group.

Boxplots were used to analyze the FDX1, CDKN2A, MTF1, and GLS genes of the patients in the two groups. FDX1 expression levels in the low-risk group of patients were significantly increased (Figure 7(a), *p* < 0.001); CDKN2A, GLS, and MTF1 expression levels were significantly increased in the high-risk group of patients (Figures 7(b)–7(d), *p* < 0.001). This shows that PHC patients with high expression of FDX1 had better prognoses, whereas those with high expression of CDKN2A, MTF1, and GLS had worse prognoses. We conducted gene set enrichment analysis to explore the potential biological functions and signaling pathways between the two groups. There were 52 active pathways in the high-risk group (Figure 8(a), FDR < 0.05). The enrichment pathways were associated with cellular metabolism, repair, growth, proliferation, apoptosis, and immunity. There were 2 active pathways were related to tricarboxylic acid metabolism in the low-risk group (Figure 8(a), FDR < 0.05).

### 3.5. Correlation between Prognostic Risk Scores and Immune Cells

**Figure 9:**
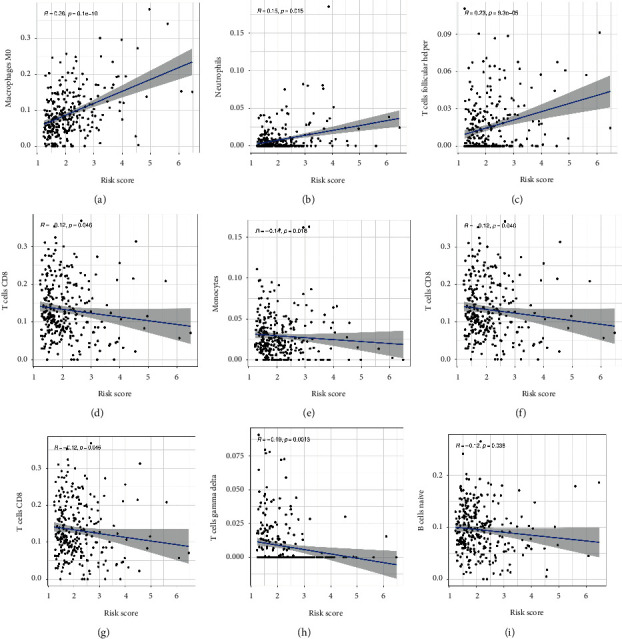
Relationships between the CRLRs and infiltration abundances of nine types of immune cells, as analyzed using Spearmen's correlation analysis. (a) M0 macrophages; (b) neutrophils; (c) follicular helper T cells; (d) resting mast cells; (e) monocytes; (f) activated NK cells; (g) CD8^+^ T cells; (h) gamma delta T cells; (i) naive B cells.

The CIBERSORT algorithm was used to analyze 22 different immune cells for the two groups of patients. There were positive correlations between the risk score and M0 macrophages (*R* = 0.36, *p*=8.1 × 10^−10^), neutrophils (*R* = 0.15, *p*=0.015), and follicular helper T cells (*R* = 0.23, *p*=9.3 × 10^−05^) (Figures 9(a)–9(c)). Negatively correlations were found between the risk score and resting mast cells (*R* = −0.23, *p*=9.9 × 10^−05^), monocytes (*R* = −0.14, *p*=0.018), activated natural killer (NK) cells (*R* = −0.13, *p*=0.027), CD8^+^ T cells (*R* = −0.12, *p*=0.046), gamma delta T cells (*R* = −0.19, *p*=0.0013), and naive B cells (*R* = −0.12, *p*=0.038) (Figures 9(d)–9(i)). This indicates that PHC prognosis is associated with immune cell infiltration.

### 3.6. Overexpression of MIR210HG, MKLN1-AS, and PLBD1-A in PHC Patients

**Figure 10:**
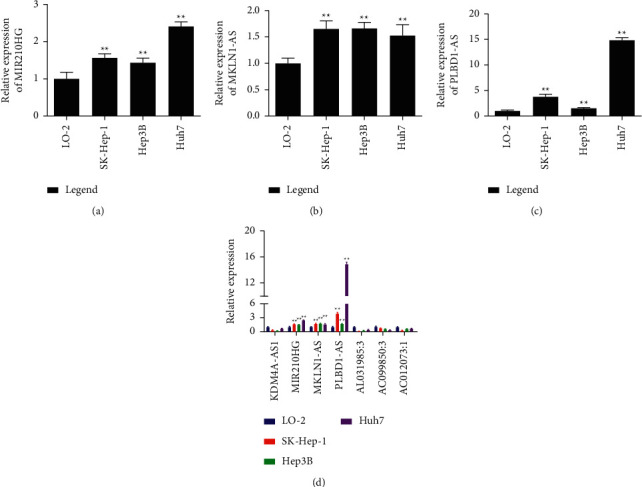
The results of RT-qPCR for (a) MIR210HG, (b) MKLN1-AS, and (c) PLBD1-AS in the hepatoma cell lines. (d) The results of RT-qPCR for the seven CRLRs in the hepatoma cell lines. ^*∗∗*^*p* < 0.01.

The expression levels of the seven CRLRs in liver cancer cells and normal hepatocytes were measured using PCR. The results showed that MIR210HG, MKLN1-AS, and PLBD1-AS were overexpressed in liver cancer cells (Figures 10(a) fig10–10(c)); the expression of the other four CRLRs was decreased. This might be attributed to the variances among the different cell lines and false-positive results of the prediction (Figure 10(d)).

## 4. Discussion

Cell death is a physiological process. The mechanism of cell death varies. Apoptosis [18], pyroptosis [19], necrosis [20], and ferroptosis [21–23] are the most commonly observed. Recently, Tsvetkov et al. found a copper-based mechanism that was completely different from the known mechanisms of cell death, and they named it “cuproptosis” [7]. LncRNAs have been shown to be associated with hepatic carcinoma [24, 25]. Therefore, we built a cuproptosis-related lncRNA prognostic risk model to predict the prognoses of patients with PHC, explore its potential pathogenesis, and facilitate individualized treatment.

Hepatoma tissue samples were divided into CRLRSM high-risk and low-risk groups. The OS of patients in the high-risk group was worse than that of patients in the low-risk group. The CRLRSM was an independent prognostic factor for PHC and had a good predictive performance for the prognoses of patients with PHC. Correlation analyses between the CRLRSM and cuproptosis-related genes showed that patients with high FDX1 expression had better prognoses, indicating that high expression of FDX1 might promote cancer cell death. patients with high expression of CDKN2A, MTF1, and GLS had worse prognoses, suggesting that CDKN2A, MTF1, and GLS might promote the proliferation of cancer cells. This is consistent with the findings of Tsvetkov et al. [7], who identified FDX1 as a key regulator of cuproptosis and an upstream regulator of protein lipoacylation, and the abundance of FDX1 and lipoacylated proteins is highly correlated with a variety of human tumors. The results also suggest that CDKN2A, MTF1, and GLS are negative feedback genes that inhibit apoptosis and promote cell survival. At the same time, the enrichment pathway of the CRLRSM high-risk group was related to tumor cell genesis and proliferation. The enrichment pathway of the CRLRSM low-risk group was mainly related to the tricarboxylic acid cycle, and was mainly in the upstream pathway entering the tricarboxylic acid cycle, indicating that the progress of the tricarboxylic acid cycle was blocked. Therefore, these seven CRLns may be potential targets for the treatment of PHC.

We validated the expression of the seven CRLRs in hepatic cancer cells and normal hepatocytes and found that MIR210HG, MKLN1-AS, and PLBD1-AS were highly overexpressed in hepatic cancer cells. Recent studies have shown that overexpression of MKLN1-AS is associated with a lower OS rate and shorter disease-free survival. The downregulation of MKLN1-AS reduces the proliferation, migration, and invasion of cancer cells and induces apoptosis. *In vivo* inhibition can also suppress the proliferation of hepatic cancer cells. MKLN1-AS has been shown to serve as a molecular sponge for miR-654-3p, upregulate the expression of HCC-derived growth factor (HDGF), and promote cancer growth [26]. The upregulation of MKLN1-AS also leads to poor prognoses in patients with PHC. MKLN1-AS positively regulates the expression of YAP1 via targeting and stabilizing YAP1 mRNA, and it enhances the proliferation, migration, and invasion of hepatic cancer cells through YAP1. It can also induce the expression of YAP1 *in vivo* to cause hepatic carcinogenesis [27].

MIR210H was first found to be overexpressed in osteosarcoma and glioma. Wang et al. discovered that hepatic cancer cells overexpressing MIR210H contributed to poor prognoses in patients, whereas silencing MIR210H inhibited proliferation, migration, and invasion of cancer cells [28]. AC099850.3 and KDM4A-AS1 are newly identified lncRNAs, and their overexpression indicates an adverse prognosis in PHC patients. Knockout of AC099850.3 might significantly inhibit the proliferative and migratory potential of hepatic cancer cells and promoted their death. A previous study proposed that AC099850.3 served as an oncogene through the PRR11/PI3K/AKT pathway [29]. KDM4A-AS1 promotes the proliferation, migration, and invasion of hepatic cancer cells *in vitro* and promotes the growth of hepatic cancer cells and lung metastasis *in vivo*. It is suggested that KDM4A-AS1 is regulated retrograde by miR-411-5p at the post-transcriptional level and promotes the expression of KPNA2 by competitively binding to miR-411-5p to activate the AKT pathway. KPN2 silencing, miR-411-5p overexpression, and AKT inhibitors (e.g., MK2206) can reverse KDM4A-AS1-enhanced hepatoma cell proliferation, migration, and epithelial-mesenchymal transformation. KDM4A-AS1 is considered to be a new hypoxia response gene that promotes the growth and metastasis of hepatic cancer [30] through KDM4A-AS1/KPNA2/HIF-1*α* signaling.

Gene set enrichment analysis showed that the mTOR, p53, ErbB, and insulin signaling pathways, ubiquitin-mediated proteolysis, inositol phosphate metabolism, and the phosphatidylinositol signaling system were associated with the metabolism, growth, proliferation, and apoptosis of cancer cells. The ubiquitin-proteasome system (UPS) had the strongest correlation. The UPS is a multicomponent system for protein degradation in cells and is involved in multiple cellular biological activities such as cell growth and differentiation, DNA replication and repair, cellular metabolism, and the immune response, affecting the degradation of most proteins in eukaryotic cells [31].UPS dysfunction has been found to be closely related to multiple diseases, including neurodegenerative diseases, cancer, cardiovascular diseases, and respiratory diseases [32].

UPS dysfunctions can be divided into two categories based on specific mechanisms. The first involves genetic mutation of the enzymes or substrates of the UPS system, in which normal UPS targets are no longer subject to being ubiquitinated and degraded; subsequently, they constantly accumulate in the cells. The second category is abnormal activation of the UPS, which accelerates the degradation of intracellular proteins. There are also UPS inhibitors, such as bortezomib, which inhibit the catalytic activity of the proteasome subunits, resulting in mitochondrial membrane depolarization and cell apoptosis. Apoptosis is induced mainly by an increase in intracellular p27 and p53 levels [33]. Second-generation proteasome inhibitors, including kafizomib and oral isazomib, are used for the treatment of multiple myeloma. TP53 encodes a transcription factor, and the tumor suppressor p53 is activated and stabilized simultaneously in response to cellular stress and DNA damage; this is the basis for its central role as a tumor suppressor [34, 35].

It has been found that the MDM2 gene is expressed in the UPS and p53 signal pathways. MDM2 inhibits p53-induced apoptosis and is the most connected functional target of p53. Its N-terminal domain binds to the transcriptional activation domain of p53, hinders the binding of p53 to its cotranscriptional activators, and subsequently inhibits the activation of p53 target genes [36]. The RING domain in the C-terminus of MDM2 has E3 ubiquitin ligase activity and can ubiquitinate and degrade p53 [37]. In some human tumors, MDM2 has been demonstrated to be upregulated abnormally due to gene magnification, increased transcription, and enhanced translation; these would induce increased degradation and decreased activity of p53 [38]. Based on the strategy of blocking the protein interaction between p53 and MDM2, some small molecules have been developed, including BI-907828 [39], milademetan [40], and APG-115 [41]. Among these agents, APG-115 and milademetan have been approved for clinical trials and have yielded preliminary clinical data [42, 43]. Upregulation of the phosphocreatine kinase signal pathway, insulin signal pathway, and ErbB signal pathway can activate the PI3K/AKT pathway and mTOR, which plays a critical role in the regulation of autophagy [44]. The mTOR signal is highly activated in most cancers, especially in the process of cell transformation, growth, and survival [45].

The correlations between risk scores and immune cells showed that immune cell infiltration is associated with the prognosis of PHC. Several enriched pathways were associated with the immune response. For example, the ErbB pathway downregulates the chemokine ligand CXCL10 through PI3K–AKT signaling and interferon regulatory factor IRF1, which results in the reduction of effector CD8^+^ T cells and recruitment of T_reg_ cells into the tumor microenvironment; this leads to immune escape and cancer growth [46]. PI3K–AKT–mTOR regulates many characteristics of the immunosuppressive microenvironment. The latest data from clinical trials and preclinical mouse models suggest that the therapeutic inhibition of the PI3K–AKT–mTOR signaling network might have dual benefits: preventing tumor progression by suppressing proliferation, migration, and survival of cancer cells, and enhancing the tumor immune surveillance pathway and intrinsic antitumor immune characteristics by inhibiting the activation of immunosuppressants [47]. Shishir et al. believed that the tumor microenvironment of HCC still remains a major challenge to therapeutic success. To explore a new strategy of combined immunotherapy will hopefully lead to major improvements in survival for patients [48].

In conclusion, hepatic carcinogenesis is a complicated process that involves multiple biological functions and pathways. This often results in unsatisfactory therapeutic outcomes and poor survival rates in patients with PHC. The seven CRLns in the high-risk group were found to be involved in multiple pathways to promote the growth, proliferation, invasion, and migration of PHC. The low-risk group was involved in multiple pathways related to the tricarboxylic acid cycle, therefore, silencing the seven CRLns may promote tumor cell death, which is a potential target for the treatment of PHC, and further research is needed to verify its mechanism and efficacy in the future. This study has several limitations. First, the data were obtained from the UCSC Xena public database, which has a limited sample size. Second, we did not explore further functional genomics and pathways, although we assessed the expression of CRLR in hepatoma cells and normal hepatocytes. Lastly, our study lacks further validation from cohort, prospective, multicenter, and real data.

## Figures and Tables

**Table 1:** PCR primer sequences.

Primer	Sequence (5′–3′)
Human-MIR210HG-F	CAGCGTTTGGAGCCTCCTGC
Human-MIR210HG-R	AGGCAACTCGGCTTGGTTATTTC
Human-KDM4A-AS1-F	CAGGTCGTGAGCGCACCCAT
Human-KDM4A-AS1-R	TCAGCCATCCAGGCAAGAGCA
Human-PLBD1-AS1-F	GTGGATTCCATCCTAGAGGCTGTG
Human-PLBD1-AS1-R	TTCCTGCTTTCTGTCCTTCATTTCAG
Human-AC099850.3-F	TCACTGCAACCTCTGCCTCCC
Human-AC099850.3-R	TTCCCTGTTGTCACTGACCTATGTAATC
Human-AL031985.3-F	CCACAAGATGCCAGCATTCA
Human-AL031985.3-R	GCCCTTGAGCCAAACGAAAC
Human-AC012073.1-F	TATGTGGAGCTGTGGTTAGTTTCC
Human-AC012073.1-R	CAAAGTGGCACTGTTCGTAATAGAC
Human-GAPDH F	AATCCCATCACCATCTTCCA
Human-GAPDH R	AAATGAGCCCCAGCCTTCT

## Data Availability

The raw data could be obtained from the corresponding author.
